# One-pass deep brain stimulation of subthalamic nucleus and ventral intermediate nucleus for levodopa-resistant tremor-dominant Parkinson’s disease

**DOI:** 10.3389/fnagi.2023.1289183

**Published:** 2023-12-21

**Authors:** Bin Liu, Junpeng Xu, Zhebin Feng, Rui Hui, Yanyang Zhang, Di Liu, Qing Chang, Xinguang Yu, Zhiqi Mao

**Affiliations:** ^1^Medical School of Chinese PLA, Beijing, China; ^2^Department of Neurosurgery, The First Medical Center of Chinese PLA General Hospital, Beijing, China

**Keywords:** tremor-dominant Parkinson’s disease, deep brain stimulation, subthalamic nucleus, ventral intermediate nucleus, levodopa response

## Abstract

**Objective:**

Tremor-dominant Parkinson’s disease (TD-PD) can be further separated into levodopa-responsive and levodopa-resistant types, the latter being considered to have a different pathogenesis. Previous studies indicated that deep brain stimulation (DBS) of the subthalamic nucleus (STN) or the globus pallidus internus (GPi) individually was not sufficient for tremor control, especially for the levodopa-resistant TD-PD (LRTD-PD). The thalamic ventral intermediate nucleus (VIM) has been regarded as a potent DBS target for different kinds of tremors. Therefore, we focused on the LRTD-PD subgroup and performed one-pass combined DBSs of STN and VIM to treat refractory tremors, aiming to investigate the safety and effectiveness of this one-trajectory dual-target DBS scheme.

**Methods:**

We retrospectively collected five LRTD-PD patients who underwent a one-pass combined DBS of STN and VIM via a trans-frontal approach. The targeting of VIM was achieved by probabilistic tractography. Changes in severity of symptoms (measured by the Unified Parkinson Disease Rating Scale part III, UPDRS-III), levodopa equivalent daily doses (LEDD), and disease-specific quality of life (measured by the 39-item Parkinson’s Disease Questionnaire, PDQ-39) were evaluated.

**Results:**

Three-dimensional reconstruction of electrodes illustrated that all leads were successfully implanted into predefined positions. The mean improvement rates (%) were 53 ± 6.2 (UPDRS-III), 82.6 ± 11.4 (tremor-related items of UPDRS), and 52.1 ± 11.4 (PDQ-39), respectively, with a mean follow-up of 11.4 months.

**Conclusion:**

One-pass combined DBS of STN and VIM via the trans-frontal approach is an effective and safe strategy to alleviate symptoms for LRTD-PD patients.

## 1 Introduction

Parkinson’s disease (PD) features four motor symptoms: tremor, bradykinesia, rigidity, and postural instability, among which tremor is the most salient manifestation, troubling about 75% of PD patients (Lees et al., 2009). Despite that resting tremor is the most common type, PD patients can also present postural and motor tremors, especially at the late stage (Abusrair et al., 2022). If tremor is the dominant complaint and disabling, this subset of PD can be categorized as tremor-dominant Parkinson’s disease (TD-PD) according to the ratio of tremor-related scores and other scores calculated from the Unified Parkinson Disease Rating Scale (UPDRS) or the Movement Disorder Society-sponsored revision (Jankovic et al., 1990; Stebbins et al., 2013). This subgroup has been deemed to suffer different pathophysiology, mainly reflected by the unmatched progressing speed and severity with other motor symptoms (Louis et al., 1999), as well as the unobvious dopaminergic neuron loss in the substantia nigra pars compacta (Paulus and Jellinger, 1991). In consequence, TD-PD patients tend to respond unsatisfactorily to dopaminergic drugs and finally resort to deep brain stimulation (DBS) surgery (Abusrair et al., 2022).

The subthalamic nucleus (STN) and the globus pallidus internus (GPi) are the two main DBS targets for TD-PD patients. STN-DBS was associated with a tremor improvement of 70–75% for PD at 1-year follow-up, and this benefit remained stable for 5 years (Benabid et al., 2009). GPi-DBS showed no significant difference in tremor improvement from STN-DBS (Wong et al., 2019). However, there still were about 10% of TD-PD patients who responded poorly to STN or GPi stimulation and this failure rate could increase to 33% when patients were evaluated under on-medication conditions (Azghadi et al., 2022). Furthermore, a retrospective study centering on PD tremor found that 33.3% (STN-DBS) and 60% (GPi-DBS) patients failed to achieve a two-point reduction of resting tremor score at 12 months postoperatively (Wong et al., 2020). These findings indicate that single STN or GPi stimulation may not be effective enough to control tremor for part of TD-PD patients.

As we all know, the ventral intermediate nucleus (VIM) in the thalamus is a classical DBS target for tremors, including resting, postural, motor, and dystonic tremors (Hariz et al., 2008; Cury et al., 2017; Iorio-Morin et al., 2020). Accordingly, TD-PD usually presents with multiple types of tremors (Selikhova et al., 2013), which indicates that stimulation of VIM seems to be preferable for TD-PD patients. However, VIM-DBS, relative to STN or GPi stimulation, showed powerless in the face of other motor symptoms of PD (Lyons et al., 2001; Hariz et al., 2008). Thus, some studies used two sets of DBS electrodes to stimulate STN (or GPi) and VIM simultaneously (Kobayashi et al., 2014; Oertel et al., 2017). Nevertheless, this scheme not only doubles the risks of bleeding and infection but increases the patients’ economic costs. Inspired by the one-pass DBS of VIM and the posterior subthalamic area in essential tremor (Barbe et al., 2018), some neurosurgical teams recently attempted to perform one-pass DBS of STN and VIM to treat TD-PD in small-sample studies (Coenen et al., 2016; Kaptan and Cakmur, 2018; Fayed et al., 2021). However, these studies neither took the dopaminergic responsiveness into account nor provided clear surgical details, such as the methods of VIM localization and three-dimensional (3D) electrode reconstruction.

Differently, we further separated TD-PD into levodopa-responsive type and levodopa-resistant type according to the levodopa challenge test (LCT). Because the treatment strategies of levodopa-resistant tremor-dominant Parkinson’s disease (LRTD-PD) are controversial in clinical practice, and the effect of DBS in this subgroup is unstable, albeit some LRTD-PD patients can still benefit from STN-DBS or GPi-DBS alone (Coenen et al., 2016). We think that this subgroup demands modified DBS surgery, for example, dual-target DBS, to guarantee the effect of DBS. Based on that, we involved five LRTD-PD patients to perform one-pass stimulation of STN and VIM, aiming to redefine the indication for this combined surgery and prove its feasibility, effectiveness, and safety. In addition, we introduce the techniques of three-tract probabilistic tractography and the 3D reconstruction of electrodes into this one-trajectory dual-target DBS scheme.

## 2 Methods

### 2.1 Subjects and rationale

All five PD patients suffering from severe drug-refractory tremor were collected at our department from July 2022 to February 2023. The inclusion criteria were as follows: (1) Diagnosis of primary PD confirmed by two experienced neurologists in movement disorder according to the MDS clinical diagnostic criteria for Parkinson′s disease (Postuma et al., 2015); (2) severe tremor (tremor is ≥3 cm in amplitude and resting tremor is present at least half of the entire examination period); (3) compromised life quality (patients have difficulty in eating and drinking on their own); (4) Disease duration ≥3 years; (5) Hoehn-Yahr stage ≥2.5; (6) UPDRS-III improvement rate of LCT < 30%; and (7) No history of other stereotactic operations. The exclusion criteria consisted of (1) Diagnosis of essential tremor plus or atypical parkinsonism syndrome; and (2) Preoperative DBS plan failed to design a suitable one-pass path to concatenate STN and VIM nuclei, usually limited by individual anatomical differences.

Demographic and baseline characteristics, such as disease duration, tremor type, medication, UPDRS score, and the 39-item Parkinson’s Disease Questionnaire (PDQ-39) were collected. We performed a standard LCT before surgery by giving Metoba equal to 1.5 times the morning levodopa equivalent dose.

### 2.2 VIM positioning and surgery planning

**Figure 1:**
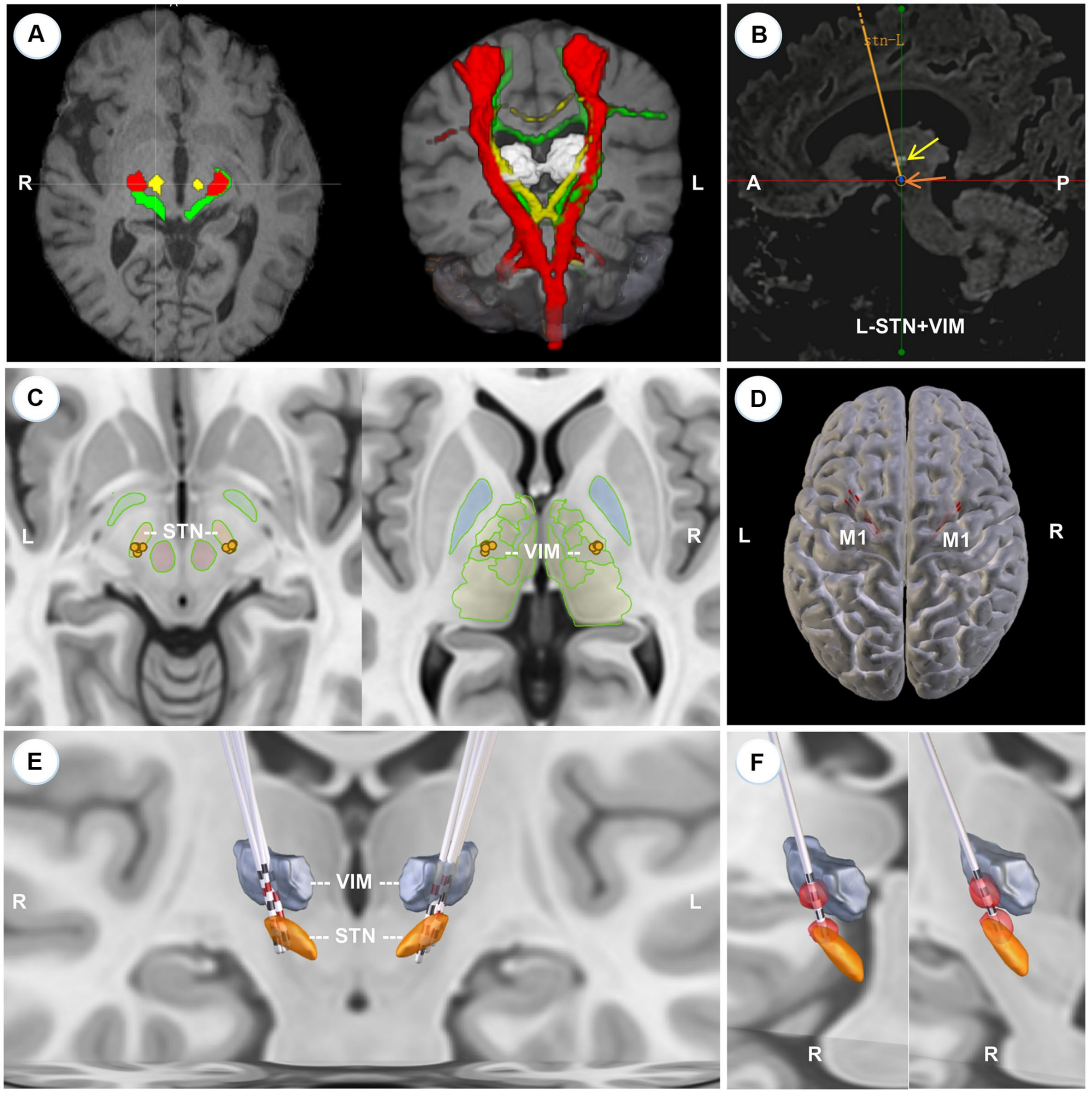
Localization of VIM, surgical planning, and 3D reconstruction of all implanted electrodes. **(A)** The axial slice (left) and the 3D model (right) of the reconstructed pyramidal tract (red), somatosensory tract (green), and DRTT (yellow) of a typical patient, superimposed on the individual T1 images. **(B)** The left-side surgical planning of a typical patient to show STN and VIM are concatenated by one trajectory in the sagittal view. The light white cluster (yellow arrow) is the mask of VIM deriving from the overlapping portion between the DRTT and the thalamus. STN is hypointense on T2 Flair images (brown arrow). **(C)** 2D illustration of the electrode position in STN (left) and VIM (right) respectively. **(D)** 3D model to show that all trajectories (red lines) are located before the primary motor cortex (M1). **(E)** 3D reconstruction of all electrodes at the group level with active contacts highlighted in red. All implanted electrodes had contacts in both STN and VIM, except two right electrodes that pass through the ventral oral posterior nucleus. **(F)** VTA models for the right two anteriorly-placed electrodes show that the two VTAs still have an overlap with the VIMs.

All patients were preoperatively scheduled for standard MR scans, including T1 images (1.0 * 1.0 * 1.0 mm^3^, no gap), Flair images (1.2 * 1.2 * 1.2 mm^3^, no gap), and diffusion images (2.0 * 2.0 * 2.0 mm^3^, b = 0 s/mm2, b = 1,000 s/mm^2^, 66 diffusion directions). Because VIM is invisible on routine MRI sequences, we performed probabilistic tractography by using the FDT tool in FSL software,^1^ via which we tracked three bundles of fibers in individual space: the pyramidal tract with the precentral gyrus as seed mask and the ipsilateral cerebral peduncle as waypoint mask, the somatosensory tract with the postcentral gyrus as seed mask and the ipsilateral thalamus as waypoint mask, and the dentato-rubro-thalamic tract (DRTT) with the dentate nucleus as seed mask and the ipsilateral superior cerebellar peduncle plus contralateral thalamus as waypoint masks. These three fibers were superimposed on the individual T1 images and the 3D model was rendered via the MRIcroGL software^2^ (Figure 1A). Considering that the decussating DRTT was more related to DBS outcomes (Deuter et al., 2022), we only tracked the decussating part of the DRTT. Besides, since the DRTT travels along the angle between the pyramidal tract and the somatosensory tract, to lower the incidence of adverse effects, the final target of VIM was identified to be at least 3 mm away from these two tracts. Additionally, we defined the overlapping portion between the thalamus and the DRTT as the mask of VIM and uploaded it to the SinoPlan system (Sinovation, Beijing, China), where the final targets and trajectories were planned. The dorsolateral portion of STN, as the primary target, was accurately identified first, followed by a backward shift of the trajectory until it penetrated through the VIM nucleus (Figure 1B). Meanwhile, the precentral gyrus, sulci, and cortical vessels should be taken into consideration before the entry point was determined. If the individual anatomical variation made it impossible to draw a one-pass trajectory, the subject would be excluded (three LRTD-PD patients were excluded).

### 2.3 Surgical procedures

On the day of surgery, a Leksell G head frame (ELEKTA, Stockholm, Sweden) was fixed on the patient’s head, followed by a CT scan that would be fused with MR images to obtain the frame coordinates. Intraoperatively, microelectrode recordings were used to further confirm the localization of both nuclei under monitored conscious anesthesia. The arrest of neuronal bursting at the same frequencies with tremor, albeit not always detectable, is a reliable clue that the microelectrode tip passed through the targets. On the condition that a long-distance high-frequency neuronal firing was not detected, the trajectory would be adjusted in light of the firing pattern and the patient’s adverse effects. Intraoperative macrostimulation was performed to observe the latent efficacy of stimulation and adverse effects by using the outer cannula of the microelectrode. Subsequently, a pair of permanent DBS electrodes with 1.5 mm contacts interval were implanted and locked bilaterally. We anticipated that the two ventral contacts were embedded into STN, and the two dorsal contacts into VIM. Finally, the implantable pulse generator was connected and implanted at the right subclavicular area subcutaneously under general anesthesia. All stereotactic plans and operations were completed by the same senior neurosurgeon who specializes in DBS surgery.

### 2.4 Leads reconstruction

Postoperatively, a CT scan was scheduled to confirm the placement of leads. The postoperative CT and preoperative MRI images of each patient were imported into the Lead-DBS software (Horn and Kuhn, 2015), where the electrodes were reconstructed in a semi-automated manner. The images were processed under the default settings, that is, coregistered and normalized with the Advanced Normalization Tools, localized with the PaCER algorithm, and rendered on the Montreal Neurological Institute (MNI) template. We chose the DISTAL Medium atlas as the reference to show the relative position between electrodes and nuclei because this atlas directly provides the 3D models of STN and VIM. Further, we simulated the volume of tissue activated (VTA) based on given stimulation parameters. Additionally, using its congeneric toolbox, Lead Group (Treu et al., 2020), we visualized the 3D model of all electrodes at the group level with the active contacts highlighted in red.

### 2.5 Programming and follow-up

One month after surgery, the DBS devices were activated by a specialized DBS programmer. First, to evaluate stimulation effects and adverse effects, we screened all contacts in a monopolar way with pulse width and frequency kept at 60us and 130 Hz separately, while voltage increased from 1 V to 4 V with a step of 0.5 V. During this process, we paid attention to how the tremor and other motor symptoms were alleviated. Second, according to the results of screening and electrode reconstruction, we activated the contact within STN and refined the stimulating parameters to see whether a monopolar stimulation could provide satisfactory tremor relief. If it could, we tended to adopt the monopolar mode, like Patient 4 who first used an STN-DBS alone (but he changed to dual-target stimulation at later follow-ups because of better tremor relief from adding VIM stimulation, see Table 1). If not, we simultaneously activated the contact within or closest to VIM to perform double monopolar or triple monopolar stimulation, like the included patients other than patient 4. In our practice, the combined stimulation of STN and VIM generally provided superior tremor control. Finally, we refined the voltage, frequency, and pulse width to maximize the DBS efficacy. Through multiple attempts, the optimal parameters were identified for chronic stimulation. During the follow-up period, the stimulation settings would be tuned when the patients felt unsatisfactory about symptom control or encountered a stimulation-related adverse effect.

**Table 1:** Demographic information and disease-related characteristics.

Patient number	Sex	Age (year)	Tremor type (resting/posture/motor)	Duration (year)	H-Y (med-off)	Pre-UPDRS-III (med-off)	Pre-UPDRS-tremor (med-off)	LCT-UPDRS-III (%)
1	M	71	+/+/+	15	2.5	48	15	20.8
2	F	72	+/+/-	12	2.5	37	13	29.7
3	M	64	+/+/+	7.5	3	52	16	25.0
4	M	62	+/+/−	8	2.5	51	15	25.5
5	M	74	+/−/+	4.5	2.5	42	13	14.3
mean	–	68.6	–	9.4	2.6	46	14.4	23.1
SD	–	5.3	–	4.1	0.2	6.4	1.3	5.8

M, male; F, female; H-Y, Hoehn-Yahr stage; UPDRS-III, the Unified Parkinson Disease Rating Scale part III; LCT, levodopa challenge test; LEDD, levodopa equivalent daily dose; SD, standard deviation.

Patients were followed regularly at 3 months, 6 months, and 12 months postoperatively. At the last follow-up, all patients were evaluated with UPDRS-III under on-medication/on-stimulation conditions. The changes in tremor, measured by items 20 and 21 of the UPDRS-III, were analyzed separately. Meanwhile, a PDQ-39 questionnaire was accomplished to show the improvement in life quality. Drugs were also documented to calculate the reduction of levodopa equivalent daily dose (LEDD). Of note, all baseline and follow-up scales were scored by the same rater.

## 3 Results

### 3.1 Baseline and demographic data

Demographic data are presented in Table 1. Five TDLR-PD Patients (four males, one female) were included in this study, with a mean age of 63.6 ± 5.3 years. All patients suffered from resting tremor and further had motor tremor or postural tremor, or both. The mean disease duration before DBS implantation was 9.4 ± 4.1 years. All patients reached Hoehn-Yahr stage 2.5 or later. The LCT improvement rates of all patients were < 30%.

### 3.2 Fibers and electrodes reconstruction

Figures 1A,B illustrates the three traced fibers and how we identified the targets and trajectories in a typical patient. As shown in Figures 1C,E, five pairs of electrodes: three sets of Medtronic 3,387 leads (Medtronic, Minneapolis, MN) and two sets of PINS L302 leads (PINS, Beijing, China), were successfully implanted into the predefined positions. All electrodes had contacts in both STN and VIM, except two right electrodes (Patient 2, Patient 3) that penetrated into the ventral oral posterior nucleus (VOP) or the VIM/VOP border zone. Even so, the simulated VTAs of these two patients overlapped with VIM partly (Figure 1F), that is, the VIM could be stimulated by the two anteriorly placed electrodes. All trajectories passed in front of the precentral gyrus (Figure 1D). The detailed position and coordinates of each contact are described in Table 2.

**Table 2:** DBS lead type and contact position.

Patient	Lead type	Right					Left				
		Contact	MNI coordinates			Location	Contact	MNI coordinates			Location
			X	Y	Z			X	Y	Z	
1	PINS-L302	1	13.0	−4.0	−4.8	STN	5	−12.2	−4.2	−4.3	STN
		2	13.9	−3.6	−1.3	STN/cZI	6	−13.2	−3.5	−1.0	STN/cZI
		3	14.7	−3.3	2.2	VOP/VIM	7	−14.2	−2.8	2.4	VOP/VIM
		4	15.4	−3.1	5.9	VIM	8	−15.0	−2.3	5.8	VIM
2	PINS-L302	1	14.0	−2.8	−2.0	STN	5	−12.9	−3.8	−3.5	STN
		2	14.7	−2.6	1.1	cZI	6	−13.8	−3.5	−0.6	STN/cZI
		3	15.2	−2.3	4.4	VOP	7	−14.7	−3.0	2.7	VIM
		4	15.7	−1.9	7.8	VOP	8	−15.5	−2.5	6.0	VIM
3	Medtronic-3387	0	13.8	−3.1	−2.3	STN	8	−13.3	−3.9	−3.8	STN
		1	14.5	−2.8	0.6	cZI	9	−13.9	−3.6	−0.9	STN/cZI
		2	15.3	−2.4	3.6	VOP	10	−14.6	−3.3	2.2	VOP/VIM
		3	16.1	−2.0	6.7	VOP	11	−15.4	−3.0	5.4	VIM
4	Medtronic-3387	0	12.1	−3.6	−4.2	STN/PSA	8	−11.0	−3.6	−6.5	SNr
		1	13.0	−3.2	−0.7	cZI	9	−11.9	−3.1	−3.0	STN
		2	13.8	−2.8	2.9	VOP/VIM	10	−12.7	−2.7	0.6	cZI/VIM
		3	14.6	−2.4	6.3	VIM	11	−13.5	−2.1	4.1	VIM
5	Medtronic-3387	0	13.6	−3.7	−5.0	STN	8	−12.5	−5.4	−5.0	STN
		1	14.3	−3.3	−1.7	STN/cZI	9	−13.6	−4.9	−1.7	STN/cZI
		2	14.9	−3.0	1.6	VOP	10	−14.6	−4.5	1.6	VIM
		3	15.3	−2.9	5.1	VIM	11	−15.5	−4.0	5.0	VIM

Coordinates represent the centers of electrode contacts in the Montreal Neurological Institute (MNI) space. All coordinates were calculated with reference to the midcommissural point (MCP). DBS, deep brain stimulation; STN, subthalamic nucleus; cZI, caudal zona incerta; SNr, substantia nigra pars reticulate; VIM, ventral intermediate nucleus; VOP, ventral oral posterior nucleus.

### 3.3 Stimulation settings

Table 3 gives an overview of stimulation settings at DBS activation and the last follow-up. Corresponding to the intention of the surgical plan (stimulating STN and VIM simultaneously), most of the patients were programmed using a double monopolar or triple monopolar setting with the implantable pulse generator working as the anode. Although Patient 4 was modulated with a monopolar scheme at first, he changed to use a double monopolar scheme at a later follow-up because of better control of tremor. The mean stimulating parameters were 2.0 ± 0.3 V (voltage), 78.2 ± 12.5 us (pulse width), and 177 ± 9.0 Hz (frequency) at the last follow-up, respectively.

**Table 3:** Stimulation parameters and adverse effects.

Patients	Time point	Stimulation parameters		Side effects
		Right	Left	
1	DBS activation	C+/3-/4-, 1.8 V, 70 us, 190 Hz	C+/6-/8-, 1.75 V, 60 us, 190 Hz	Transient Dizziness
	LFU	C+/3-/4-, 1.6 V, 70 us, 190 Hz 4+/1-, 2.05V, 70 us, 190 Hz (interleaving)	C+/5-/8-, 1.6 V, 70 us, 190 Hz	–
2	DBS activation	C+/1-/3-, 2.0 V, 60 us, 180 Hz	C+/5-/6-, 2.2 V, 60 us, 180 Hz	Transient Dizziness
	LFU	C+/1-/3-, 2.0 V, 60 us, 180 Hz	C+/5-/6-, 2.2 V, 60 us, 180 Hz	–
3	DBS activation	C+/0-/2-, 2.4 V, 90 us, 170 Hz	C+/9-/10-/11-, 2.0 V, 80 us, 170 Hz	–
	LFU	C+/0-/2-, 2.2 V, 90 us, 170 Hz	C+/9-/10-/11-, 2.0 V, 80 us, 170 Hz	Dystonia
4	DBS activation	C+/1-, 2.1 V, 90 us, 170 Hz	C+/9-, 1.8 V, 90 us, 170 Hz	Numbness
	LFU	C+/1-/3-, 1.8 V, 90 us, 170 Hz	C+/9-/11-, 1.8 V, 90 us, 170 Hz	–
5	DBS activation	C+/1-/3-, 2.2 V, 60 us, 130 Hz	C+/9-/10-/11-, 2.3 V, 60 us, 130 Hz	–
	LFU	C+/1-/3-, 2.0 V, 90 us, 170 Hz	C+/9-/10-/11-, 2.0 V, 90 us, 170 Hz	–

DBS, deep brain stimulation; LFU, last follow-up.

### 3.4 Effects of STN/VIM DBS

Table 4 documents the changes in symptoms, life quality, and LEDD contributed by simultaneous STN and VIM stimulation during follow-up. The mean follow-up duration was 11.4 months. The severity of all motor symptoms (UPDRS-III) yielded a mean improvement of 53% at the last follow-up. For tremors specifically, a more significant improvement (82.6%) was achieved. Additionally, the quality of life (PDQ-39) presented a mean improvement of 52.1%. However, the LEDD did not reduce significantly.

**Table 4:** Changes in severity of symptoms, quality of life, and medication.

Patients	Mean follow-up duration (month)	UPDRS-III			UPDRS-tremor			PDQ39			LEDD (mg)		
		Baseline (med-on)	LFU (med-on, DBS-on)	Improvement (%)	Baseline (med-on)	LFU (med-on, DBS-on)	Improvement (%)	Baseline (med-on)	LFU (med-on, DBS-on)	Improvement (%)	Baseline	LFU	Improvement (%)
1	15	38	23	55.3	13	2	84.6	44	23	47.7	650	650	0
2	14	26	15	42.3	10	3	70.0	63	24	61.9	525	400	23.8
3	12	39	18	53.8	12	0	100.0	59	20	66.1	450	450	0
4	10	38	17	55.3	12	3	75.0	53	29	45.3	825	450	45.5
5	6	36	15	58.3	12	2	83.3	33	20	39.4	400	400	0
Mean	11.4	35.4	17.6	53.0	11.8	2	82.6	50.4	23.2	52.1	570	470	17.5
SD	3.6	5.4	3.3	6.2	1.1	1.2	11.4	12.1	3.7	11.4	170.8	103.7	20.5

UPDRS, the Unified Parkinson Disease Rating Scale; PDQ-39, the 39-item Parkinson's Disease Questionnaire; LEDD, levodopa equivalent daily dose; LFU, last follow-up; SD, standard deviation.

### 3.5 Adverse effects

No surgery-related or device-related adverse events occurred. As documented in Table 3, the stimulation-related adverse effects in the course of follow-up were transient dizziness, limb dystonia, and numbness, which were generally transient or subsided by adjustment of stimulation parameters or change of active contacts.

## 4 Discussion

In this article, we presented five LRTD-PD patients who experienced a DBS surgery targeting both STN and VIM simultaneously via one trans-frontal trajectory. All five pairs of long-interval (1.5 mm) electrodes were successfully implanted into the predefined positions without surgery-derived adverse events. Through optimizing stimulation settings, all five patients obtained a significant improvement whether in movement severity (especially in tremor severity) or quality of life.

### 4.1 Why does the combined DBS of STN and VIM work?

The “finger–switch–dimmer” model indicated that PD tremor is induced by dysfunction of the basal ganglia, facilitated in the thalamus, and modulated by the cerebellum (Helmich et al., 2012; Duval et al., 2016). Thus, the pathogenesis of PD tremor is involved in the basal ganglia and cerebello-thalamo-cortical (CTC) circuit (Helmich et al., 2012; Duval et al., 2016). Stimulation of the key nodes of these circuits should be effective for PD tremor. For STN specifically, as a hub node of basal ganglia, the effectiveness of its DBS on PD motor symptoms has been verified by thousands of clinical practices. As for its relationship with tremors, electrophysiological studies monitored neuronal high-frequency oscillations synchronized with limb tremors within STN (Levy et al., 2000), and STN stimulation at near-tremor frequency could entrain limb tremors in PD patients (Cagnan et al., 2014). However, STN-DBS is often considered to be powerful for resting tremor but less so for postural and motor tremors (Iorio-Morin et al., 2020). Similar to that of STN, electrophysiological monitoring identified the “tremor cells” bursting at the same frequency as tremors in the thalamus, and most of these cells were located in VIM (Lenz et al., 1988). As a key point of DRTT (part of CTC), VIM has been proven to be the optimal site within the thalamus for stereotactic interventions to treat various tremors, especially the postural and motor tremors (Iorio-Morin et al., 2020; Chandra et al., 2022). Nevertheless, it is powerless against other PD motor symptoms (Lyons et al., 2001; Hariz et al., 2008). Taken together, stimulating STN and VIM simultaneously has a synergistic effect on PD symptoms, with STN stimulation targeting the bradykinesia, rigidity and resting tremor, and VIM stimulation controlling other kinds of tremors.

### 4.2 What can we learn from previous studies?

A handful of case reports and case series used this one-trajectory combined DBS of STN and VIM to treat TD-PD patients. Kaptan and Cakmur (2018) first provided a technical report describing the feasibility of one-pass stimulation of STN and VIM by a trans-frontal approach. However, they did not report the curative effect and displayed the exact location of each contact. Fayed et al. (2021) used this trans-frontal one-pass dual-target approach to treat TD-PD patients who underwent a 38.2% mean improvement in overall UPDRS-III scores and a 59% mean improvement in tremor-related scores. By contrast, our cases yielded a 53% overall UPDRS-III improvement and an 82.6% mean tremor improvement. We speculate that the difference in DBS efficacy arises from the factor that part of their cases were implanted unilaterally and stimulated with a monopolar mode (only STN was activated), which may indicate the superiority of bilateral DBS and dual-target stimulation. In addition, the trans-parietal approach was also adopted to concatenate STN and VIM. Coenen et al. (2016) reported two TD-PD patients treated by the one-pass combined DBS of STN and DRTT via a trans-parietal approach. One of them underwent STN-DBS surgery first but received unsatisfactory tremor control. However, after removing the previous STN leads due to infection and undergoing another combined DBS of STN and DRTT, he received an obvious tremor relief (78% improvement in tremor items). This case also indicates the superiority of combined stimulation of STN and VIM. However, all these previous studies did not distinguish between levodopa-sensitive and levodopa-resistant TD-PD, which is an important factor to consider when choosing the one-pass dual-target DBS scheme, as Coenen et al. also believed (Coenen et al., 2016).

### 4.3 Why do we just focus on the LRTD-PD subgroup?

Although the effect of STN-DBS is complex and has not been clarified, several preclinical studies have found that STN-DBS could increase striatal dopamine release and metabolism (Pazo et al., 2010; Yamamoto et al., 2014). Moreover, the fact that STN-DBS has an apparent strength in reducing dopaminergic medication after surgery has been widely accepted (Kleiner-Fisman et al., 2006; Bove et al., 2021). These findings suggest there should exist a latent link between STN-DBS and the dopaminergic system. Hence, for those levodopa-responsive TD-PD cases, we hold that STN-DBS is the first choice to control tremors, which is also consistent with our clinical impression. Instead, excessive pursuit of more complex surgical operations, like the one-trajectory dual-target DBS, would bring in more potential risks and problems (see below).

On the other hand, for levodopa-resistant cases, a single STN-DBS is not sufficient to ensure effective relief of motor symptoms. A ≥ 30% improvement rate in LCT had been acknowledged as a criterion to determine whether PD patients could acquire obvious benefits from DBS surgery (Saranza and Lang, 2021). Moreover, LRTD-PD may not have a dopaminergic pathogenetic basis (Madelein van der Stouwe et al., 2020). A combined EMG-functional MRI study indicated that relative to levodopa-sensitive TD-PD, LRTD-PD arises from a larger contribution of the cerebellum (Dirkx et al., 2019). Other neuroimaging studies also confirmed the involvement of the cerebellum (van den Berg and Helmich, 2021). This means that the output of the cerebellum works more in the pathogenesis of LRTD-PD. VIM, as the main relay of the efferent fibers of the cerebellum, had better be further stimulated for LRTD-PD patients. Therefore, we refined the indication for this combined DBS surgery and focused only on the LRTD-PD subgroup.

In addition, a lower LEDD reduction (only 17.5%) was detected in our TD-PD patients (Table 4), compared with the result of Fayed et al. (2021) who reported a 40.2% LEDD reduction. The main reason is that we only included LRTD-PD patients, while they included all TD-PD patients regardless of the levodopa responsiveness. For LRTD-PD patients, increasing dosages could not bring corresponding benefits because of their dopamine-resistant properties, so they tended to take lower doses of drugs preoperatively. After DBS surgery, considering that the patients were already on a low dose of medication, we just slightly reduced their drug intake or advised them to keep similar dose as before. Therefore, it is the low preoperative drug dose that leads to the low improvement in LEDD.

### 4.4 How to localize VIM more accurately and safely?

To date, VIM localization has relied mainly on indirect techniques, like the coordinate-based method based on the anterior and posterior commissure, which tends to be inaccurate due to the individual anatomical variation (Anthofer et al., 2014; Parras et al., 2022). Albeit advanced imaging methods such as quantitative susceptibility mapping sequences, fast gray matter acquisition T1 inversion recovery sequences, and high-field MRI sequences increased the visibility of VIM in the thalamus, these sequences present low reliability and have not been popularized around the world (Lehman et al., 2020). Currently, MR diffusion tractography has developed into the most promising technique to directly identify the VIM area. However, previous tractography studies were mostly based on deterministic tractography (Feltrin et al., 2022), which showed limited capability of delineating complex fibers or crossing fibers such as DRTT (Yang et al., 2022). Even though some researchers used the approach of probabilistic tractography, most of they just tracked one tract (DRTT) to localize the VIM region, instead of three tracts (Lehman et al., 2020). Differently and more precisely, we tracked three bundles of fibers: pyramidal tract, somatosensory tract, and DRTT by using probabilistic tractography (see the Methods), not only delineating the location of VIM but reducing the risks of adverse effects resulting from overflowed stimulation of the internal capsule laterally and the ventral caudal nucleus posteriorly. This may to some extent explain why the incidence of adverse effects of our patients was lower than that of other combined DBS reports.

### 4.5 What should we look for in this surgery?

Although this dual-target DBS surgery can significantly improve tremor control, some caveats should be noted. Firstly, the achievement of the trans-frontal approach is demanding. Since VIM is located further posteriorly and superiorly relative to STN, the entry point needs to be moved backward. Limited by the primary motor cortex, lateral ventricles, and cortical or intraparenchymal vessels, the surgical design of concatenating VIM and STN could likely not be completed. Thus, not every patient is suitable for a trans-frontal approach, which entails careful patient selection before surgery. Secondly, the trajectory of the trans-frontal approach usually punctures into the premotor area. The edema or hemorrhage around the entry points could lead to limb weakness, which, even if absent in our cases, was reported in one patient by Fayed et al. (2021). This is also one of the reasons why we recommend this DBS protocol only for LRTD-PD and not for all TD-PD. Finally, owing to the anatomical constraint, the electrodes might pass through the anterior part of the VIM and enter the VOP, like in two of our cases (Figures 1C,E). Despite that, the reconstructed VTAs had a large overlap with the VIMs (Figure 1F). In fact, no significant difference existed in the number of “tremor cells” between VOP and VIM (Kobayashi et al., 2003), and stimulation of VOP or the VOP/VIM border zone was also effective in controlling tremor (Yamamoto et al., 2004; Foote et al., 2006). Beyond that, the thickness of VIM is only 2–3 mm approximately in the anterior–posterior direction, so the electrode is best placed near the anterior border of VIM to prevent current from spreading to the thalamic somatic sensory nucleus (the ventral caudal nucleus) to induce contralateral paresthesias (Benabid et al., 1996). Hence, we consider it reasonable to place the electrodes into VOP or the VOP/VIM border if placement into VIM is difficult.

### 4.6 Limitations

This study still holds some limitations. Firstly, the sample size is small, which partly results from our strict patient selection (only LRTD-PD). The small sample size impeded us from correlating the improvement rates with the overlapping volume between VTAs and nuclei. As an exploratory study, we encourage larger-sample studies or prospective randomized controlled trials to validate the safety and effectiveness of this one-pass combined DBS of STN and VIM. Secondly, although we looked through the effect of each contact individually when the DBS devices were activated about 1 month after surgery, we did not observe the effects of chronic stimulation of VIM only or STN only, except for Patient 4 who was given monopolar stimulation of STN for 3 months and changed to double monopolar mode at subsequent follow-up because of better tremor control. In the future, a crossover paradigm, for example, VIM or STN stimulation for 3 months in turn, followed by combined stimulation, should be attempted to detect target-specific outcomes and verify the superiority of dual-target DBS. Finally, the follow-up duration is short, and outcomes at longer follow-ups need to be reported.

## 5 Conclusion

Overall, one-pass combined stimulation of STN and VIM via the trans-frontal approach is a feasible, safe, and effective alternative to control refractory PD tremor, especially for the LRTD-PD subtype. In the future, larger-scale studies or crossover-designed randomized controlled trials are warranted to validate the superiority of this one-trajectory dual-target DBS.

## Data availability statement

The original contributions presented in the study are included in the article/supplementary material, further inquiries can be directed to the corresponding authors.

## Ethics statement

The studies involving humans were approved by Medical Ethics Committee of Chinese PLA General Hospital. The studies were conducted in accordance with the local legislation and institutional requirements. The participants provided their written informed consent to participate in this study.

## Author contributions

BL: Data curation, Formal analysis, Investigation, Methodology, Writing – original draft. JX: Data curation, Methodology, Writing – original draft. ZF: Writing – review & editing, Investigation. RH: Data curation, Methodology, Writing – review & editing. YZ: Investigation, Writing – review & editing, Software. DL: Writing – review & editing, Formal analysis. QC: Writing – review & editing, Formal analysis. XY: Writing – review & editing, Conceptualization, Supervision. ZM: Writing – review & editing, Conceptualization, Funding acquisition.

## Footnotes

^1^ https://fsl.fmrib.ox.ac.uk/fsl/fslwiki/FSL

^2^ https://www.nitrc.org/projects/mricrogl
